# Characterization of Panton–Valentine leukocidin-positive *Staphylococcus aureus* from skin and soft tissue infections and wounds in Nigeria: a cross-sectional study

**DOI:** 10.12688/f1000research.15484.1

**Published:** 2018-07-30

**Authors:** Olayemi O. Ayepola, Nurudeen A. Olasupo, Louis O. Egwari, Frieder Schaumburg

**Affiliations:** 1 Department of Biological Sciences, Covenant University, Ota, Ogun, Nigeria; 2 Department of Microbiology, Lagos State University, Ojo, Lagos, Nigeria; 3 Institute of Medical Microbiology, University Hospital Münster, Münster, Germany

**Keywords:** Staphylococcus aureus, PVL

## Abstract

***Background:*** *Staphylococcus aureus* is a significant pathogen implicated in numerous nosocomial and community-acquired infections. The Panton–Valentine leukocidin (PVL) can be associated with severe necrotizing diseases such as pneumonia, skin and soft tissue infection (SSTI).

**Methods**: In total, 96 *S. aureus *isolates were obtained from patients presenting with wounds (n=48) and soft tissue infections (SSTIs, n=48). These were characterized based on their antimicrobial susceptibility profile, the possession of virulence genes (e.g. capsular type, PVL), accessory gene regulator ( *agr*) type, and the staphylococcal protein A ( *spa*) type. The production of the PVL protein was assessed by western blotting.

**Results**: All isolates were susceptible to methicillin. The resistance was highest to penicillin (97.9%), followed by trimethoprim/sulfamethoxazole (85.4%) and tetracycline (10.4%). The PVL gene was found in 83.3% of isolates from SSTIs and in 79.2% of isolates from wound. Of these, 53 (68%) produced PVL as assessed by western blotting. The most prevalent *spa *type was the t084 (78.1%, n=75) and, majority of the isolates carried *agr*2 (82.3%, n=79).

**Conclusions**: Prevalence of antibiotic resistant PVL-positive methicillin susceptible *S. aureus* strains has severe implications on PVL mediated infections.

## Introduction

*Staphylococcus aureus* is an important human pathogen that causes significant hospital and community acquired infections ^1^. *S. aureus* producing Panton-Valentine leukocidin (PVL) is linked to a broad array of necrotizing diseases such as pneumonia and skin and soft tissue infections (SSTIs) ^2^. PVL is more frequently associated with community isolates ^3^. PVL is a pore-forming toxin that can kill myeloid cells by forming channels in the plasma membrane, leading to loss of osmotic balance that ultimately lyses the cel ^4^. Earlier reports have shown PVL to be one of the most important virulence determinants in *S. aureus* from sub Saharan Africa ^5^. This study was conducted to investigate the presence of virulence genes including *lukS*-PV/ *lukF-*PV, the production of the PVL protein and the antibiotic resistance in methicillin-susceptible *S. aureus* strains isolated from wounds and SSTIs between 2010 and 2011.

## Methods

### Ethical statement

Ethical approval for this study was obtained from the Ethics Committee of the Department of Biological Sciences, Covenant University, Ota, Ogun State, Nigeria (CUNG-2010-035). All participants signed a written informed consent before the commencement of the study.

### Characterization of isolates

In this study we made use of an already existing database which has been published ^6^. The study was conducted in four health facilities in Ogun and Lagos States of Nigeria between June 2010 and May 2011. Samples were collected from patients presenting with SSTIs and wound infections. The isolation and identification of the isolates were done by culture and genotyping. A total of 96 *S. aureus* isolates were obtained from wounds (n=48) and SSTIs (n=48). The Vitek automated systems (bioMérieux, Marcy L’Étoile, France) was employed to determine the antibiotic susceptibility profile. The PVL gene ( *lukS-*PV */lukF-*PV), capsular polysaccharides ( *cap 5, cap 8*), exfoliative toxins ( *eta, etb*), the toxic shock syndrome toxin ( *tst*) and the *agr* type were detected by PCR. All amplifications was done in a thermocycler (Bio-Rad, Munich, Germany). The cycling conditions and primers used are as earlier published. Detection of the *lukS-*PV */lukF-*PV gene was carried out using primer sequences: *luk-PV-1*(5'-ATCATTAGGTAAAATGTCTGGACATGATCCA-3') and *luk-PV-2* (5' GCATCAASTGTATTGGATAGCAAAAGC- 3') ^7^. The negative control was *S. aureus* ATCC 49230 (MSSA) and the positive control was sta 635/636 (a PVL-positive CA-MRSA strain). Primers specific for the variable segment of the *cap* locus. Cap5-f: (5'-GAAAGTGAACGATTAGTAGAA-3') Cap5-r: (5'-GTACGAAGCGTTTTGATAGTT-3') Cap 8-f: (5'-GTGGGATTTTTGTAGCTTTT-3') Cap 8-r: (5'-CGCCTCGCTATATGAACTAT-3') was used for the capsular typing ^8^. Sequences specific for exfoliative toxins; *eta*, *etb* and the toxic shock syndrome toxin; *tst* were detected by multiplex PCR ^9^. The *agr* types of the *S. aureus* strains were determined by the multiplex PCR strategy ^10^. Extracellular production of PVL by *lukS-*PV */lukF-*PV –positive strains was evaluated by a Western blot using in-house antibodies raised in rabbits (anti- *lukF-PV*: 334 µg/ml, anti- *lukS-PV*: 900 µg/ml ^11^. The nitrocellulose membrane (Schleicher & Schüll, Dassel, Germany) was first incubated with rabbit anti- *lukS*- *PV/lukF-PV* antibodies (in-house antibodies, 1:1000 in TBST) and later incubated with polyvalent goat alkaline-phosphatase-conjugated anti-rabbit antibodies (1:1000 in TBST, DAKO, Germany, D0487). The membranes were washed and the bands visualized using alkaline phosphatase color development substrate (BCIP/NBT, Thermo Fischer Scientific, 34042) ^11^. The production of PVL was determined semi-quantatively in four categories: no PVL production; low PVL production, high and very high PVL production. The genetic diversity of all isolates was determined by the staphylococcal protein A ( *spa*) typing ^12^. The highly polymorphic region X of the protein A gene, which is composed of a variable number of 24-bp repeats, was amplified by PCR. s *pa* types were determined with the Ridom StaphType software version 1.5 beta (Ridom GmbH, Würzburg, Germany). All statistical computations were performed in SPSS Version 25. Data is explored using relevant descriptive analysis alongside chi ^2^ to measure any association between antibiotic resistance, virulence genes and *lukS-*PV */lukF-*PV. P<0.05 is deemed to be statistically significant.

Results of Vitek assay, PCR results for virulence genes, agr typing and spa typing Click here for additional data file. Copyright: © 2018 Ayepola OO et al. 2018 Data associated with the article are available under the terms of the Creative Commons Zero "No rights reserved" data waiver (CC0 1.0 Public domain dedication).

Results of PCR experiments. Gel photo for amplification of lukS-pv and lukF-pv gene Click here for additional data file. Copyright: © 2018 Ayepola OO et al. 2018 Data associated with the article are available under the terms of the Creative Commons Zero "No rights reserved" data waiver (CC0 1.0 Public domain dedication).

Results of PCR experiments. Gel photo for amplification of agr group Click here for additional data file. Copyright: © 2018 Ayepola OO et al. 2018 Data associated with the article are available under the terms of the Creative Commons Zero "No rights reserved" data waiver (CC0 1.0 Public domain dedication).

## Results and discussion

We analyzed the characteristics of the PVL-positive *S. aureus* isolates as well as the relationship between antibiotic resistance, virulence genes and PVL gene ( Table 1). Antibiotic resistance was highest to penicillin (100% in SSTI isolates and 94% in wound isolates), followed by trimethoprim/sulfamethoxazole (84% in SSTI isolates and 83% in wound isolates) and tetracycline (8% in SSTI isolates and 10% in wound isolates ( Table 1). This is consistent with an earlier study which showed similar resistance rates for penicillin (98%), trimethoprim/sulfamethoxazole (80%) and tetracycline (18%) in Nigeria ^6^. All isolates were methicillin-susceptible. The *lukS-*PV */lukF-*PV gene was detected in 83.3% (n=40) of SSTI isolates and 79.2% (n=38) of wound isolates. Reports from other African countries have shown high rates of PVL positive MSSA ranging from 17% to 74% ^5^. For example, a study in an Algiers hospital reported a prevalence of 72% among clinical isolates ^13^. A multi-center study reported that deep-seated SSTIs associated with the PVL gene resulted in more hospitalizations of patients and this led more often to incision and drainage ^14^. A meta-analysis showed PVL to be consistently associated with SSTIs than invasive diseases ^15^. In a study carried out in Gabon, PVL-positive isolates were found to occur more in SSTIs, and PVL was also associated with resistance to trimethoprim/sulfamethoxazole ^16^

**Table 1.** Association between *PVL* gene and antibiotic resistance.

Antimicrobial resistance		*PVL* Gene		OR (95%CI)	P value
		Absent	Present		
		Count (%)	Count (%)		
Penicillin	R	16 (17.0)	78 (83.0)	0.04 (0.002–0.9)	0.003
	S	2 (100.0)	0		
Oxacillin	R	2 (100.0)	0	23.8 (1.1–519.2)	0.003
	S	16 (17.0)	78 (83.0)		
Gentamicin	R	4 (100.0)	0	48.7 (2.5–955.2)	<0.001
	S	14 (15.2)	78 (84.8)		
Levofloxacin	R	4 (100.0)	0	48.7 (2.5–955.2)	<0.001
	S	14 (15.2)	78 (84.8)		
Tetracycline	R	5 (50.0)	5 (50.0)	5.6 (1.4–22.2)	0.007
	S	13 (15.1)	73 (84.9)		
Trimethoprim/ sulfamethoxazole	R	12 (14.6)	70 (85.4)	0.23 (0.1– 0.8)	0.012
	S	6 (42.9)	8 (57.1)		
*cap 8*	Absent	5 (100.0)	0	63.96 (3.3–1226)	<0.001
	Present	13 (14.3)	78 (85.7)		
*cap 5*	Absent	13 (14.3)	78 (85.7)	0.02 (0.001–0.3)	<0.001
	Present	5 (100.0)	0		
*spa* type	t064	1 (100.0)	0	<0.000	<0.001
	t084	11 (14.7)	64 (85.3)		
	t159	1 (100.0)	0		
	t194	1 (100.0)	0		
	t2304	0	6 (100.0)		
	t8435	0	4 (100.0)		
	t8441	3 (100.0)	0		
*agr*	agr1	5 (100.0)	0	NA	NA
	agr2	12 (15.2)	67 (84.8)		
	agr4	1 (8.3)	11 (91.7)		

Note: R=resistant, S=susceptible

**Figure 1.**
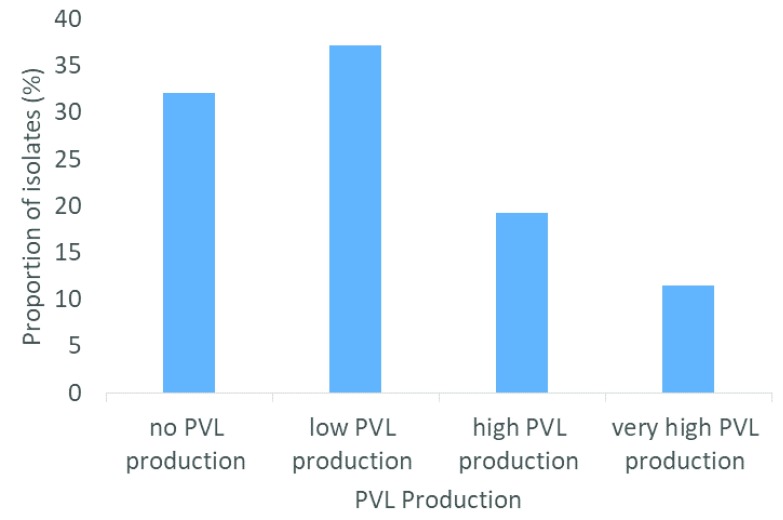
Quantification of Panton-Valentine leukocidin (PVL) production in PVL-positive *S. aureus* isolates.

The presence of the PVL gene does not necessarily guarantee that the protein will be expressed and, if it is, toxin levels could vary widely from strain to strain. The production of PVL (in contrast to the sole presence of *lukS-*PV */lukF-*PV) was observed in 75% of *lukS-*PV */lukF-*PV SSTI isolates and 60.5% of *lukS-*PV */lukF-*PV wound isolates. *In vitro* variation in the production of PVL by different strains of *S. aureus* has been reported and this suggests important differences in transcriptional and/or translational control of gene expression ^17^. In this study, the level of PVL produced by *lukS-*PV */lukF-*PV positive *S. aureus* isolates varied from strain to strain ( Figure 1). It was observed in that none of the PVL-positive strains harboured other toxin genes such as *eta, etb* and *tst*. Seven different *spa* types were identified ( Table 1). The most prevalent *spa* type was t084 (78.1%, n=75). An earlier study revealed a significant association of the spa-CC 084 PVL-positive isolates with PVL-positive isolates ^6^. Typing of the *agr* locus, which controls the expression of many *S. aureus* virulence factors, showed that most isolates (82.3%, n=79) possessed the *agr*2, while none carried *agr*3. Other studies have linked isolates carrying an *agr*4 allele to exfoliatin-related diseases and usually carry *eta* and/or *etb* ^18, 19^. These were absent in this study.

In conclusion, this study showed that many *S. aureus* isolates in Nigeria carry the PVL genes but few produced PVL *in vitro*. Antibiotic resistance combined with the presence of the PVL genes, has serious implications in the treatment of *S. aureus* infections. This study is limited by the few study locations. A larger study population is needed to provide a better understanding of the clones of *S. aureus* in Nigeria. The results is however significant for regional surveillance.

## Data availability

The data referenced by this article are under copyright with the following copyright statement: Copyright: © 2018 Ayepola OO et al.

Data associated with the article are available under the terms of the Creative Commons Zero "No rights reserved" data waiver (CC0 1.0 Public domain dedication). 

Dataset 1: Results of Vitek assay, PCR results for virulence genes, *agr* typing and *spa* typing. 10.5256/f1000research.15484.d211827 ^20^

Dataset 2: Results of PCR experiments. Gel photo for amplification of *lukS-pv* and *lukF-pv* gene. 10.5256/f1000research.15484.d211828 ^21^

Dataset 3: Results of PCR experiments. Gel photo for amplification of *agr* group. 10.5256/f1000research.15484.d211829 ^22^

The results were previously presented at the 4th International Conference on Prevention & Infection Control (ICPIC 2017) Geneva, Switzerland. 20–23 June 2017. Antimicrobial Resistance and Infection Control 2017, 6(Suppl 3):52. DOI 10.1186/s13756-017-0201-4. Poster 261.

## References

[1] Holmes A Ganner M McGuane S: *Staphylococcus aureus* isolates carrying Panton-Valentine leucocidin genes in England and Wales: frequency, characterization, and association with clinical disease. *J Clin Microbiol.* 2005;43(5):2384–90. 10.1128/JCM.43.5.2384-2390.2005 15872271 PMC1153723

[2] Tristan A Bes M Meugnier H: Global distribution of Panton-Valentine leukocidin--positive methicillin-resistant *Staphylococcus aureus*, 2006. *Emerg Infect Dis.* 2007;13(4):594–600. 10.3201/eid1304.061316 17553275 PMC2725977

[3] Bocchini CE Hulten KG Mason EO Jr: Panton-Valentine leukocidin genes are associated with enhanced inflammatory response and local disease in acute hematogenous *Staphylococcus aureus* osteomyelitis in children. *Pediatrics.* 2006;117(2):433–40. 10.1542/peds.2005-0566 16452363

[4] Yoong P Torres VJ: The effects of *Staphylococcus aureus* leukotoxins on the host: cell lysis and beyond. *Curr Opin Microbiol.* 2013;16(1):63–69. 10.1016/j.mib.2013.01.012 23466211 PMC3670676

[5] Schaumburg F Alabi AS Peters G: New epidemiology of *Staphylococcus aureus* infection in Africa. *Clin Microbiol Infect.* 2014;20(7):589–596. 10.1111/1469-0691.12690 24861767

[6] Ayepola OO Olasupo NA Egwari LO: Molecular Characterization and Antimicrobial Susceptibility of *Staphylococcus aureus* Isolates from Clinical Infection and Asymptomatic Carriers in Southwest Nigeria. *PLoS One.* 2015;10(9):e0137531. 10.1371/journal.pone.0137531 26348037 PMC4562701

[7] Lina G Piémont Y Godail-Gamot F: Involvement of Panton-Valentine leukocidin-producing *Staphylococcus aureus* in primary skin infections and pneumonia. *Clin Infect Dis.* 1999;29(5):1128–1132. 10.1086/313461 10524952

[8] Goerke C Esser S Kümmel M: *Staphylococcus aureus* strain designation by *agr* and *cap* polymorphism typing and delineation of *agr* diversification by sequence analysis. *Int J Med Microbiol.* 2005;295(2):67–75. 10.1016/j.ijmm.2005.01.004 15969467

[9] Becker K Friedrich AW Lubritz G: Prevalence of genes encoding pyrogenic toxin superantigens and exfoliative toxins among strains of *Staphylococcus aureus* isolated from blood and nasal specimens. *J Clin Microbiol.* 2003;41(4):1434–9. 10.1128/JCM.41.4.1434-1439.2003 12682126 PMC153929

[10] Lina G Boutite F Tristan A: Bacterial competition for human nasal cavity colonization: role of Staphylococcal *agr* alleles. *Appl Environ Microbiol.* 2003;69(1):18–23, (accessed July 14, 2018). 10.1128/AEM.69.1.18-23.2003 12513972 PMC152380

[11] Löffler B Hussain M Grundmeier M: *Staphylococcus aureus* panton-valentine leukocidin is a very potent cytotoxic factor for human neutrophils. *PLoS Pathog.* 2010;6(1):e1000715. 10.1371/journal.ppat.1000715 20072612 PMC2798753

[12] Mellmann A Friedrich AW Rosenkötter N: Automated DNA sequence-based early warning system for the detection of methicillin-resistant *Staphylococcus aureus* outbreaks. *PLoS Med.* 2006;3(3):e33. 10.1371/journal.pmed.0030033 16396609 PMC1325475

[13] Ramdani-Bouguessa N Bes M Meugnier H: Detection of methicillin-resistant *Staphylococcus aureus* strains resistant to multiple antibiotics and carrying the Panton-Valentine leukocidin genes in an Algiers hospital. *Antimicrob Agents Chemother.* 2006;50(3):1083–5. 10.1128/AAC.50.3.1083-1085.2006 16495274 PMC1426459

[14] Alabi A Kazimoto T Lebughe M: Management of superficial and deep-seated *Staphylococcus aureus* skin and soft tissue infections in sub-Saharan Africa: a post hoc analysis of the StaphNet cohort. *Infection.* 2018;46(3):395–404. 10.1007/s15010-018-1140-6 29667040

[15] Shallcross LJ Fragaszy E Johnson AM: The role of the Panton-Valentine leucocidin toxin in staphylococcal disease: a systematic review and meta-analysis. *Lancet Infect Dis.* 2013;13(1):43–54. 10.1016/S1473-3099(12)70238-4 23103172 PMC3530297

[16] Kraef C Alabi AS Peters G: Co-detection of Panton-Valentine leukocidin encoding genes and cotrimoxazole resistance in *Staphylococcus aureus* in Gabon: implications for HIV-patients’ care. *Front Microbiol.* 2015;6:60. 10.3389/fmicb.2015.00060 25699036 PMC4318419

[17] Hamilton SM Bryant AE Carroll KC: *In vitro* production of panton-valentine leukocidin among strains of methicillin-resistant *Staphylococcus aureus* causing diverse infections. *Clin Infect Dis.* 2007;45(12):1550–8. 10.1086/523581 18190315

[18] Jarraud S Lyon GJ Figueiredo AM: Exfoliatin-producing strains define a fourth *agr* specificity group in *Staphylococcus aureus*. *J Bacteriol.* 2000;182(22):6517–22. 10.1128/JB.182.22.6517-6522.2000 11053400 PMC94802

[19] Jarraud S Mougel C Thioulouse J: Relationships between *Staphylococcus aureus* genetic background, virulence factors, *agr* groups (alleles), and human disease. *Infect Immun.* 2002;70(2):631–41. 10.1128/IAI.70.2.631-641.2002 11796592 PMC127674

[20] Ayepola OO Olasupo NA Egwari LO: Dataset 1 in: Characterization of Panton–Valentine leukocidin-positive *Staphylococcus aureus* from skin and soft tissue infections and wounds in Nigeria: a cross-sectional study. *F1000Research.* 2018 10.5256/f1000research.15484.d211827 PMC6171726 30345027

[21] Ayepola OO Olasupo NA Egwari LO: Dataset 2 in: Characterization of Panton–Valentine leukocidin-positive *Staphylococcus aureus* from skin and soft tissue infections and wounds in Nigeria: a cross-sectional study. *F1000Research.* 2018 10.5256/f1000research.15484.d211828 PMC6171726 30345027

[22] Ayepola OO Olasupo NA Egwari LO: Dataset 3 in: Characterization of Panton–Valentine leukocidin-positive *Staphylococcus aureus* from skin and soft tissue infections and wounds in Nigeria: a cross-sectional study. *F1000Research.* 2018 10.5256/f1000research.15484.d211829 PMC6171726 30345027

