# Supplementary figures and images for: Characterization of Panton–Valentine leukocidin-positive Staphylococcus aureus from skin and soft tissue infections and wounds in Nigeria: a cross-sectional study
Source: F1000Res. 2018 Jul 30;7:1155. [Version 1] doi: 10.12688/f1000research.15484.1 (PMC6171726; doi:10.12688/f1000research.15484.1)

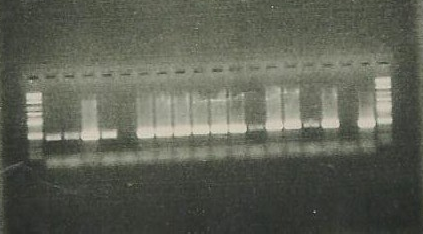

Supplement: Results of PCR experiments. Gel photo for amplification of lukS-pv and lukF-pv gene [file f1000research-7-16878-s0001.tgz › 524fa926-2029-42e9-a26b-e024bbda1ac2_Dataset_2.tif]

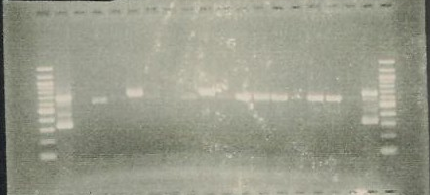

Supplement: Results of PCR experiments. Gel photo for amplification of agr group [file f1000research-7-16878-s0002.tgz › 35593923-a22b-45b9-9147-c97b9e70d064_Dataset_3.tif]
