# Supplementary material for: Characterization of Panton–Valentine leukocidin-positive Staphylococcus aureus from skin and soft tissue infections and wounds in Nigeria: a cross-sectional study
Source: F1000Res. 2018 Jul 30;7:1155. [Version 1] doi: 10.12688/f1000research.15484.1 (PMC6171726; doi:10.12688/f1000research.15484.1)
Supplement: Peer review history

# Peer review: Referee response for version 1

**DOI:** 10.5256/f1000research.16878.r38327

**Published:** 2018-09-27

**Authors:** Funmilola A. Ayeni

**Affiliations:** 1 Department of Pharmaceutical Microbiology, Faculty of Pharmacy, University of Ibadan, Ibadan, Nigeria

**Overview**

 The authors characterised PVL phenotypically and also investigate the presence of gene coding for PVL production, antibiotic susceptibility and agr production. The study is interesting as it goes further from detection of PVL gene to semi quantify the product **.**

**Introduction**

 Line 3, remove first `and` or recast in ``such as pneumonia and skin and soft tissue infections``

 Spa typing is not part of the stated objectives.

**Method**

**Characterization of isolates**

 ``In this study we made use of an already existing database which has been published ^6^.`` It should be clearly stated the data used from the referenced study above.

 The Method section should be rewritten so that all previously described method in Ayepola et al (2015) should not be rewritten in the present article but only referred to.

**Results and discussion**

 It will be interesting to also expatiate on the proportion of isolates that are PVL positive molecularly and phenotypically i.e. the isolates with the genes and also producing PVL and those with the genes without PVL.

 Spa typing reported in this study should be discussed too in relation to its epidemiological significance.

I have read this submission. I believe that I have an appropriate level of expertise to confirm that it is of an acceptable scientific standard.

# Peer review: Referee response for version 1

**DOI:** 10.5256/f1000research.16878.r37150

**Published:** 2018-09-13

**Authors:** Solayide Abosede Adesida

**Affiliations:** 1 Department of Microbiology, Faculty of Science, University of Lagos, Lagos, Nigeria

**Title**

 The *PVL production* did not appear to *correlate with* the presence of the gene. Therefore, the title should be modified to reflect the variability concerning the production of pvl, its detectable level, presence of the pvl genes and resistance rates.

**Abstract**

 The authors investigated 96 *S. aureus *isolates were obtained from patients presenting with wounds and soft tissue infections in four health facilities in two States in Nigeria. Resistance to penicillin, trimethoprim/sulfamethoxazole was more than 80% and all isolates were susceptible to methicillin. The PVL gene was found in 83.3% and 79.2% of isolates from SSTIs and wound respectively. 53 (68%) produced PVL by western blotting. The most prevalent *spa *type was the t084 (78.1%, n=75) and, majority of the isolates carried *agr*2 (82.3%, n=79). The objective (s) of the study was not stated.

**Introduction**

 Change “cel” to “cell”

**Methods**

1. There should be comma after the statement “In this study”
2. Since the isolates have been described as part of a larger study, in my opinion, extensive presentation of the previous methods used for characterising the isolates is not required. The current emphasis should be on the core findings which have not been presented in the existing or published database (ref. 6). I suggest, you present a table summarizing the characteristics of the 96 isolates as obtained in the database. 

**Results and discussion**

 Information on pvl production and the presence of the genes is limited for the region under surveillance. In my opinion, this aspect of the work is relatively novel. Therefore, kindly, reconcile your findings on level of pvl production and the presence of the genes. You may also correlate these with the antibiotic resistance and perhaps the other results in the database. However, there is a lot more reports on S. aureus from skin and soft skin infection (wounds) with reference to pvl genes/production than you have cited. Please, see Sharma-Kuinkel et al ^1^, Zhang et al, 2018, www.nature.com/scientific reports, Hamilton et al. Clinical Infectious Diseases, 2007, 45 (2007). Other studies in Nigeria should also be considered to enrich the discussion. Overall, the discussion should be revised appropriately. However, I approve subject to the corrections suggested.

I have read this submission. I believe that I have an appropriate level of expertise to confirm that it is of an acceptable scientific standard.

## References

[1] Sharma-KuinkelBKAhnSHRudeTHZhangYTongSYRuffinFGenterFCBraughtonKRDeleoFRBarriereSLFowlerVG: Presence of genes encoding panton-valentine leukocidin is not the primary determinant of outcome in patients with hospital-acquired pneumonia due to Staphylococcus aureus. *J Clin Microbiol*.2012;50(3) : 10.1128/JCM.06219-11 848-56 10.1128/JCM.06219-11 22205797PMC3295120
